# Technological mediation and humanization of nursing care: a systematic literature review

**DOI:** 10.15649/cuidarte.3537

**Published:** 2024-06-26

**Authors:** Clara Inés Padilla García, Isabel Jiménez Becerra

**Affiliations:** 1 Escuela de Enfermería, Universidad Industrial de Santander, Bucaramanga, Colombia. Estudiante de Doctorado en Educación y Sociedad, Universidad de La Salle, Bogotá, Colombia. E-mail: cipadiga@uis.edu.co Universidad Industrial de Santander Escuela de Enfermería Universidad Industrial de Santander Bucaramanga Colombia cipadiga@uis.edu.co; 2 Coordinadora Subsistema de Investigación, Ciencia, Cibercultura y Tecnosociedad, Directora de Tesis, Doctorado en Educación y Sociedad, Universidad de La Salle, Bogotá, Colombia. E-mail: ijimenez@unisalle.edu.co Universidad de la Salle Universidad de La Salle Bogotá Colombia ijimenez@unisalle.edu.co

**Keywords:** Social Skills, Nursing Care, Nursing, Education, Technology, Habilidades Sociales, Cuidados de Enfermería, Enfermería, Educación, Tecnología, Habilidades Sociais, Cuidados de Enfermagem, Enfermagem, Educado, Tecnologia

## Abstract

**Introduction:**

One of the purposes of nursing education is to provide the health system with highly competent professionals oriented to the generation of humane care practices in their daily work. To achieve this purpose, it is essential to identify the needs that arise within the teaching processes and to clearly establish how the pedagogical use of technologies can improve learning environments.

**Objective:**

To investigate and critically evaluate the contribution of technology to the strengthening of the humanization of care in the field of nursing.

**Materials and Methods:**

The methodological approach for systematic literature reviews defined by Okoli, which involves following a rigorous and standardized process to systematically and explicitly identify, evaluate, and synthesize the existing body of research. Initially, 51 articles were selected for analysis. After applying exclusion criteria, 26 studies were extracted and reviewed, identifying categories that highlight the positive influence of technology on cognitive, psychomotor, and affective competencies. Subsequently, the document with the main conclusions was drafted.

**Results:**

The findings reveal the effectiveness of various technological environments in nursing education, highlighting the prioritization of competencies linked to knowing and doing. However, there is an observed tendency to underestimate affective competencies crucial for humane care.

**Discussion:**

The results revealed a diverse landscape regarding the impact of various technologies on the development of nursing competencies, highlighting both strengths and limitations. The ability of these tools to create immersive and realistic learning environments is emphasized, although the need to delve into competencies that promote humane care is acknowledged.

**Conclusions:**

Future research is required to understand the contribution of technologies to the knowledge, attitudes, and values of the professional in training to promote humane nursing care.

## Resumen

**Introducción:**

Uno de los propósitos de la formación en enfermería es proveer al sistema de salud de profesionales altamente competentes y orientados a la generación de prácticas de cuidado humanizadas desde su quehacer cotidiano. Para lograr este propósito es fundamental identificar las necesidades que surgen dentro de los procesos de enseñanza y establecer claramente cómo el uso pedagógico de las tecnologías puede mejorar los entornos de aprendizaje.

**Objetivo:**

Indagar y evaluar críticamente el aporte de la tecnología al fortalecimiento de la humanización del cuidado en el campo de la Enfermería.

**Materiales y Métodos:**

Enfoque metodológico para revisiones sistemáticas de literatura definido por Okoli, que implica seguir un proceso riguroso y estandarizado para identificar, evaluar y sintetizar de manera sistemática y explícita el cuerpo existente de investigaciones. Se seleccionaron inicialmente 51 artículos para su análisis. Tras aplicar los criterios de exclusión, se extrajeron y revisaron 26 estudios identificando las categorías que destacan la influencia positiva de la tecnología en las competencias cognitivas, psicomotoras y afectivas. Posteriormente se ejecutó la redacción del documento con los principales hallazgos.

**Resultados:**

los hallazgos revelan la efectividad de diversos ambientes tecnológicos en la formación de enfermería, destacando la priorización de competencias vinculadas con el conocer y hacer. Sin embargo, se observa una tendencia a subestimar las competencias afectivas cruciales para el cuidado humanizado.

**Discusión:**

Los resultados revelan un panorama diverso sobre el impacto de diversas tecnologías en el desarrollo de competencias en enfermería, destacando tanto fortalezas como limitaciones. Se enfatiza la capacidad de estas herramientas para crear entornos de aprendizaje inmersivos y realistas, aunque se reconoce la necesidad de profundizar en competencias que fomenten el cuidado humanizado.

**Conclusión:**

Se requiere de investigaciones futuras para comprender el aporte de las tecnologías en el saber ser, actitudes y valores del profesional en formación, para promover un cuidado de enfermería humanizado.

## Resumo

**Introdujo:**

Uma das finalidades da formado em enfermagem é dotar o sistema de saúde de profissionais altamente competentes e orientados para a geração de práticas de cuidado humanizadas no seu cotidiano de trabalho. Para atingir este propósito, é fundamental identificar as necessidades que surgem nos processos de ensino e estabelecer claramente como o uso pedagógico das tecnologias pode melhorar os ambientes de aprendizagem.

**Objetivo:**

Investigar e avaliar criticamente a contribuido da tecnologia para o fortalecimento da humanizado do cuidado na área da Enfermagem.

**Materiais e Métodos:**

Abordagem metodológica para revisóes sistemáticas da literatura definida por Okoli, que envolve seguir um processo rigoroso e padronizado para identificar, avaliar e sintetizar de forma sistemática e explícita o corpo de pesquisa existente. Foram selecionados inicialmente 51 artigos para análise. Após aplicado dos critérios de exclusão, foram extraídos e revisados 26 estudos, identificando as categorias que destacam a influencia positiva da tecnologia nas competencias cognitivas, psicomotoras e afetivas. Posteriormente, foi elaborado o documento com as principais conclusoes.

**Resultados:**

os achados revelam a efetividade dos diversos ambientes tecnológicos na formado em enfermagem, destacando a priorização de competencias vinculadas ao saber e ao fazer. Contudo, há uma tendencia a subestimar as competencias afetivas cruciais para um cuidado humanizado.

**Discussáo:**

Os resultados revelam um panorama diversificado sobre o impacto das diversas tecnologias no desenvolvimento de competencias de enfermagem, destacando pontos fortes e limitações. Enfatiza-se a capacidade dessas ferramentas de criar ambientes de aprendizagem imersivos e realistas, embora se reconheça a necessidade de se aprofundar em competencias que promovam um cuidado humanizado.

**Conclusáo:**

Sáo necessárias futuras pesquisas para compreender a contribuido das tecnologias nos conhecimentos, atitudes e valores dos profissionais em formado, para promover o cuidado de enfermagem humanizado.

## Introduction

The use of information and communication technologies (ICT) enhances learning and promotes the construction of knowledge^1^. Re-evaluating the roles of students, teachers, and society and adopting a creativity, humanization, and critical thinking approach is crucial^2^. In the 21st century, the competencies to adapt to change and to develop as citizens are fundamental, covering cognitive, psychomotor, and affective areas^3^. Developing these competencies involves acquiring technical skills and the ability to approach challenges with empathy and ethics; it requires self-directed technological models^1^ that, inspired by the "techno-society,"^4^ train empathetic individuals capable of mapping problems and intervention scenarios.

Nursing requires comprehensive training with a socio-formative approach^5^ that includes aspects of personal and professional development^6^. ICT has proven to be useful for citizenship education^7^^)^ and social empathy^4^. Such training has been improved by technology-based learning^8^^, ^^9^, but the integration of cognitive, psychomotor, and affective aspects is needed. According to Watson^10^, nursing education has excluded the emotional and affective component, requiring an educational reorientation that promotes knowledge and "respect for patient’s dignity, uniqueness, individuality and humanity"^11^ through the development of competent professionals^12^. This implies the use of technology with a greater focus on the patient^13^, taking into account the learning ecologies^14^ and the networked society^15^. In this scenario, teachers need to promote pedagogical change^16^ to design learning environments^4^ that adapt learning experiences to students^2^. This study aims to examine and critically evaluate the contribution of technology to the strengthening of the humanization of care, inviting us to re-evaluate the formative aspects linked to the essence of "self" to provide humane care.

## Materials and Methods

**Figure 1:**
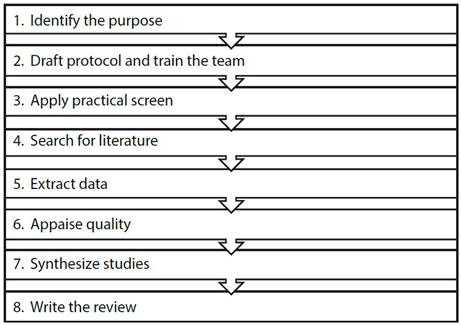
Guide to conducting a systematic review of the literature

*Source: Adaptedfrom Okoli*^17^

A standalone systematic literature review was conducted using Okoli's approach (Figure 1). All collected data is available in Mendeley Data for free access and consultation^18^.

Table 1 details the steps of the standalone systematic literature review, and Table 2 presents the concepts of competencies and technological mediation used.

**Table 1:** Steps of the standalone systematic literature review

Step	Description
Identify the purpose	The purpose of this systematic literature review was to explore and evaluate the contribution of technology in strengthening the humanization of nursing care. To achieve this goal, the following question was posed: What is the contribution of
Draft protocol and train	educational technology in strengthening the humanization of care among nursing students? Two researchers conducted the review. After defining the research question, they detailed each step and specified how the information would be extracted. During this process, the researchers established the definitions of competencies and technology
the team	environments. Once the search process was designed, researchers were trained in
Apply practical screen	proper note-taking, instrument definition, and effective review techniques. At this point, several criteria were considered for inclusion in the review, related to: a) publication period between 2020 and March 2023; b) articles published in journals; c) publications in English and Spanish; d) studies conducted in the medical, nursing,
Search for literature	and social sciences fields; e) studies conducted in educational and health institutions with nursing students; and f) open access documents. The search strategy consisted of exploring academic databases, including Scopus, Web of Science, and the PubMed search engine. These databases were selected based on criteria such as indexing, ease of navigation, access to data, and impact factor. Once the search was complete, the researchers chose Mendeley Reference Manager software
Extract data	for information management and record-keeping to ensure the efficient organization of the data collected. Figure 2 shows a summary of the texts found and the combined search of terms in the databases. The review and data extraction of the retrieved articles were systematically conducted by two researchers with expertise in technological mediation and nursing care. An analysis matrix was used to facilitate the collection of key information related to technology and its impact on education, addressing guiding questions such as: what is the problem under investigation? What is the general object of the research? What
Appraise quality	is the scientific positioning? What is the methodological approach and study design? What are the results and contributions? What are the conclusions? One researcher reviewed the articles for relevant information, while the other supervised and verified the process to ensure quality and consistency. Researchers limit the quality assessment to quantitative studies: randomized controlled trials and quasi-experimental studies published in Q1, Q2, and Q3 journals. The evaluation criteria were rigorous study design, control groups use, randomization, use of statistics, and theoretical basis. The existence of informed consent, privacy
Synthesize studies	protection, and reliable data management were verified. These criteria were detailed and recorded in a table for a more precise and systematic assessment of the quality of the studies analyzed. Based on the information gathered, we proceeded to analyze, organize, and compare the data obtained from each of the selected articles. The analysis began with an evaluation of the various technological mediations used in the studies, such as simulation, gamification, virtual reality, and others. Based on the findings, two main categories of analysis were identified: cognitive and psychomotor competencies and affective competencies.

**Table 2:** Technological mediation and competency concepts

Concept	Description
Competency	It encompasses a set of abilities developed through processes that lead people to become competent to perform multiple activities (social, cognitive, cultural, affective, work, productive), through which they project and demonstrate their ability to solve a problem within a specific and changing context^3^.
Cognitive Competency	They represent a combination of attributes related to knowing, understanding, and knowing how to act practically and operationally in actual clinical situations^3^.
Psychomotor Competencies	They determine the nurses' work dimension and involve performing specific procedures, techniques, and skills necessary to provide safe and competent care^3^.
Affective Competencies	They are related to the dimension of the 'self,' attitudes, and values3, and include the ability to understand and manage one's own and others' affections and feelings, including empathy, emotional sensitivity, and the ability to express and regulate emotions appropriately^6^.
Technological mediation	Using information and communication technologies as tools that facilitate the construction of personalized learning experiences adapted to students needs, allowing the creation of innovative teaching environments and promoting student autonomy^1^.

**Figure 2:**
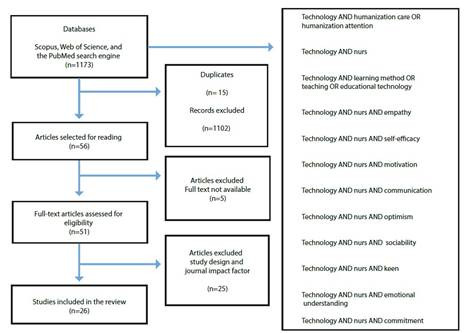
Article search and selection process

Figure 2 shows the diagram of the systematic literature review.

Table 3 shows the quality criteria and distribution of the studies reviewed.

**Table 3:** Reviewed studies distribution

Criterion	Percentages by criterion		
Study design	53.84% - (Experimental)	46,15 % - (Cuasiexperimentales)	N/A
Impact	73.07% - (Q1)	46.15 % -	7,69% - (Q3)
Geographical location*	42.30 % - (Taiwan (6) and Turkey (5))	15.38% - (South Korea (2) and Spain (2))	30.76 % - (China (1), Malta Arabia (1), Singapore (1), Egypt (1), France (1))

* 11.54 of the articles do not specify a geographic location.

## Results

The information on technological environments and competencies is presented in Table 4, which allows for quick identification and comparison of the data from the selected studies.

**Table 4:** Technological mediation and competencies

Author	Technological mediation	Cognitive competencies	Psychomotor competencies	Affective competencies
Nadler et al., 2022^19^	High-fidelity clinical simulation vs. conventional teaching	Knowledge		Satisfaction
Tseng et al., 2021^20^	Simulation and information technology integration vs. conventional teaching		Practical and physical skills	
Kim et al., 2021^21^	Non-contact practice session with smart technology vs. conventional practice session		Practical skills	
Üzen et al., 2020^22^	Standardized patient vs. high-fidelity manikin vs. Partial task trainer	Knowledge	Skills	Stress
Craig et al., 2021^23^	High-fidelity simulation vs. conventional teaching	Knowledge		Self-confidence
Alkhalaf & Wazqar, 2022^24^	High-fidelity simulation vs. traditional learning labs		Technical skills	
Kurt & Öztürk, 2021^25^	Mobile Augmented Reality (MAR) vs. conventional teaching	Knowledge		Motivation Self-confidence
Yildiz & Demiray, 2022^26^	Virtual reality (VR) vs. intravenous injection arm model		Technical skills	
Öz & Ordu, 2021^27^	Web-based education and Kahoot vs. conventional teaching	Knowledge	Technical skills	
Chang et al., 2022^28^	Online game-based learning with the watchsummarize- question approach vs. video-based learning	Learning achievement		Self-efficacy Learning engagement Satisfaction
Avşar et al., 2023^29^	Traditional teaching and reinforcement using gamification vs. conventional teaching and evaluation	Knowledge		
Blanié et al., 2020^30^	Combining simulation with gamification vs. conventional teaching	Clinical reasoning		Satisfaction Motivation
Zhu et al., 2021^20^	Massive Online Open Courses (MOOCs) vs. conventional teaching	Self-directed learning Critical thinking		Self-efficacy
Nisar et al., 2022^32^	Electronic training programme (e-training) vs. Specialist-led conventional teaching	Therapeutic Assessment		Self-efficacy Attitudes and beliefs Satisfaction
Chang et al., 2021^33^	Virtual simulation-based, mobile technology application vs. Conventional teaching with printed materials	Knowledge Cognitive load	Technical skills	Satisfaction
Yilmaz et al., 2022^34^	Infrared technology vs. traditional teaching	Knowledge	Technical skills	
Jang & Suh, 2022^35^	Mobile-based, multimedia, nursing competency evaluation system vs. text-based conventional evaluation	Knowledge		Satisfaction
Rueda et al., 2022^36^	Non-face-to-face teaching with passive training and multimedia system vs. face-to-face teaching with active training in a simulation scenario		Tasks and procedures compliance	Satisfaction
Chang et al., 2022^37^	Knowledge-based Chatbot System vs. conventional, image- and video-based teaching	Academic performance Critical thinking		Satisfaction
Jiménez et al., 2021^38^	Virtual simulation-based training			Emotional understanding Self-efficacy Optimism Sociability Affection
Hwang et al., 2022^39^	Virtual simulation vs. conventional teaching	Learning achievement		Self-efficacy Communication
Chen et al., 2020^40^	Simulation and educational videos vs. conventional teaching	Knowledge	Assessment skills	Empathy
Rodríguez et al., 2022^41^	Augmented reality (AR) vs. conventional teaching	Knowledge Understanding		Attention Motivation Autonomous learning
Lo et al., 2022^42^	Immersive virtual reality training vs. conventional teaching with 2D video	Knowledge Cognitive load		Satisfaction Motivation
Grech & Grech, 2021^43^	Gamified educational webinar vs. non‐gamified webinar			Engagement Interaction
Elzeky et al., 2022^44^	Gamified flipped classroom vs. traditional flipped classrooms	Knowledge Intensity of preparation		Motivation Self-confidence

The data were categorized into nursing education competencies, and abstracts of the articles are presented in Table 5.

**Table 5:** Characteristics of the selected articles

Author	Objetive	Method/design/sample	Description/type of mediation used	Result	Journal’s impact level	Country
Kim et al., 2021^21^	To assess the impact of highfidelity clinical simulation on undergraduate teaching, specifically in the Pediatric Nursing area.	Quasi-experimental pre- and post-test study Total: 93 nursing students EG: 46 CG: 47	Training program on children’s healthcare in clinical conditions and complications in hospital settings. EG: High-fidelity clinical simulation CG: Conventional teaching	The mean difference between the preand post-test knowledge was 4.04 points (p=0.0004) higher among the EG participants. The EG obtained a higher mean difference between the knowledge pre- and post-tests (by 3.89 points, p=0.0075) than that obtained by CG. In relation to the satisfaction scale, high scores were achieved with simulation experiences.	Q3	Brazil
Tseng et al., 2021^20^	To determine the impact of combining clinical simulation scenario training and Information Technology Integrated Instruction (ITII) on teaching nursing skills.	Purposive sampling experimental study Total: 120 nursing students EG: 61 CG:59	Medical-surgical nursing teaching program. EG: Simulation and information technology CG: Conventional teaching	There was a significant difference in course grades between the two groups after the intervention (Year 4) [t(61.59) = 2.392, p = 0.018, Cohen’s d = 0.46]. For the lab scores, the results indicated that the EG’s average scores were significantly higher than the CG’s average by 3.46 points [t (61.58) = 1.944, p = 0.048, Cohen’s d = 0.36]. For the clinical internship scores, the results showed no significant differences between the two groups, with the EG outperforming the CG by only 0.04 points.	Q1	Taiwan
Kim et al., 2021^21^	To develop non-contact CPR training using Smart technology for nursing students and examine its effects, focusing on the accuracy of their performance.	Prospective, singleblind, randomized, and controlled trial Total: 64 nursing students EG: 31 CG: 33	CPR training program. EG: Smart technology (realtime feedback) CG: conventional teaching	Overall EG's CPR performance scores significantly increased by 14.13 points right after training and slightly decreased by 2.36 at 4 weeks later, compared to the CG's, which increased by 9.45 points and then decreased by 5.09. The EG significantly improved in the accuracy of CPR, mouth-to-mouth ventilation, and ability of CPR performance compared to the CG.	Q2	South Korea
Uzen et al., 2020^22^	To compare the effect of different simulation modalities on knowledge, skill, stress, satisfaction, and self-confidence levels of students receiving undergraduate education in three nursing schools.	Randomized, controlled experimental study 139 nursing students Standardized patient: 48 High-fidelity manikin: 45 Partial task trainer: 46	Nursing education program focused on internal medicine nursing. Standardized patient High-fidelity manikin Partial task trainer	After the practices, post-test results of knowledge levels of the three groups were found to be similar (F = 1.48, p = 0.231). There was a significant difference between the skill scores of the students that were assessed during the practice (p < .05). In the practice which was performed with the standardized patient, the skill scores of the students were significantly lower during the practice compared with high fidelity and partial task trainer (p = .001). After the practice, the stress level of the standardized patient group was significantly higher than that of the other two groups (p < .05).	Q1	Not reported
Craig et al., 2021^23^	To examine the effects of an educational strategy that includes medication safety enhancement (MSE) simulations on the medication administration knowledge, competency, and confidence levels of undergraduate nursing students learning this process.	Quasi-experimental replication study Total: 80 nursing students EG: 35 CG: 45	Educational strategy for learning safe medication administration. EG: High-fidelity simulation CG: Conventional teaching	For the medication safety knowledge assessment, it was found that both groups saw an increase in mean score from baseline to week 4: from M = 16.94 to M = 18.45 (an increase of 1.52) for EG and from M = 17.18 to M = 17.82 (an increase of 0.64) for CG. The intervention implemented in EG positively impacted on the participants' selfconfidence compared to CG, although the differences were not significant in all the elements assessed.	Q1	United States
Alkhalaf & Wazqar, 2022^24^	To investigate the effects of high-fidelity simulation (HFS) technology on the competency of nursing students in the management of chemotherapy extravasation (ECMC) and the transfer of this skill from traditional learning labs to clinical settings.	Non-randomized quasi-experimental study (TREND) Total: 68 nursing students EG: 34 CG: 34	Training program on chemotherapy extravasation management. EG: High-fidelity simulation CG: Traditional learning labs	CG participants attained a lower ECMC competency level in managing extravasation in the traditional learning lab (μ′ = 17.47) than EG (μ′ = 17.91). In the chemotherapy daycare unit, the ECMC competency of participants who did not receive high-fidelity simulation training (μ′= 18.38) was lower than that of those who received high-fidelity simulation training (μ′=19.53). The improvement in ECMC competency between the traditional learning lab (μ′=17.91) and the clinical setting (μ′ = 19.53) was slightly increased for EG participants (+1.62) compared to CG (+0.91) from the traditional learning lab (μ′= 17.47) to clinical setting (μ′= 18.38), suggesting that high-fidelity simulation training does not enhance the transfer of skill to the patient care.	Q1	Saudi Arabia
Kurt & Öztürk, 2021^25^	To evaluate the effect of Mobile Augmented Reality (MAR) educational materials on nursing students' knowledge and skill levels on injection practices.	Experimental study with a control group 122 first-year nursing students EG: 64 CG: 58	Training on injection practices. EG: Mobile Augmented Reality CG: conventional teaching	Post-knowledge test scores were statistically significant as they were higher in EG (79.61) than in CG students (41.52). 68.8% of the EG students stated that their motivation to learn increased, 64.1% said that their self-confidence improved, and 54.7% stated that their fear of the injection practice procedure decreased.	Q1	Turkey
Yildiz & Demiray, 2022^26^	To determine the effect of using virtual reality technology in nursing student training for intravenous catheterization and fluid delivery.	Experimental study/ Randomized controlled trial Total: 56 nursing students EG: 29 CG: 27	Nursing student training for intravenous catheterization and fluid delivery. EG: Virtual reality CG: Intravenous injection arm model	The score of the EG students was 88.94 ± 9.22 (min: 68.12- max: 100), and that of the CG students was 65.13 ± 11.12 (min: 48.13-max: 87.50). A statistically significant difference was found between the total skill scores of the EG and CG students (p = 0.001).	Q2	Turkey
Öz & Ordu, 2021^27^	To review the effects of Kahoot usage within the framework of web-based education evaluation regarding nursing students' intramuscular injection (IM) knowledge and skills.	Quasi-experimental design Total: 110 nursing students EG: 51 CG: 59	Fundamental Principles and Applications in Nursing II Course. EG: Web-based education and Kahoot usage CG: Conventional teaching	EG had significantly higher mean knowledge scores (M = 7.4; SD = 1.4) than CG (M = 5.4; SD = 1.8). The EG had significantly higher mean scores in skill performance (M=29.5; SD=30) than the CG (M=25.4; SD=16.6).	Q1	Turkey
Chang et al., 2022^28^	To assess the effect of integrating online game-based learning with the watch-summarizequestion strategy on improving nursing students' learning achievement, self-efficacy, learning engagement, and learning satisfaction in sputum suction skill training.	Quasi-experimental study with pre- and post-test design Total: 45 nursing students EG: 21 CG: 24	Clinical nursing course on sputum suction skill training. EG: Online gamebased learning with the watchsummarize- question strategy CG: Video-based learning	In the EG, learning achievement and selfefficacy had respective adjusted averages of 90.97 (standard error = 2.14) and 4.74 (standard error = 0.17), compared with 64.40 (standard error = 2.26) and 3.84 (standard error = 0.21) for the CG. The independent samples t-test showed that the score of the EG was higher than that of the CG for learning engagement (t = 2.11, p < .05) and learning satisfaction (t = 1.73, p < .05).	Q1	Taiwan
Avşar et al., 2023^29^	To evaluate the effect of reinforcement using the Gimkit game and question-and-answer method on the achievement test scores of nursing students.	Quasi-experimental model using the pretest-posttest control group model Total: 95 students EG: 48 CG: 47	First-year nursing course. EG: Gamification reinforcement CG: conventional teaching and assessment	The difference in the pre-test and post-test mean scores was 28.17 in the EG and 19.76 in the CG. As a result of the independent sample t-test, a statistical difference was found between the two groups (t = 2.66, p = 0.009).	Q1	Turkey
Blanie et al., 2020^30^	To compare the respective educational value of simulation using serious game and a traditional teaching method to improve clinical reasoning skills necessary to detect patient deterioration by nursing students.	Experimental study/ randomized controlled trial Total: 146 Nursing students EG:73 CG:73	Early detection of signs of clinical deterioration and interprofesional communication in a clinical setting. EG: simulation by gaming CG: traditional teaching	The script concordance tests (SCT) scores were 59 ± 9 in the EG (n = 73) and 58 ± 8 in the CG (n = 73) (p = 0.43). One month later, the SCT scores were 59 ± 10 in the EG (n = 65) and 58 ± 8 in the CG (n = 54) (p = 0.77). Following the training session, all students said that their knowledge of the different steps of the clinical reasoning process had increased. The scores were all above 3.4/5, with no significant difference between groups. Global satisfaction and motivation were highly valued in both groups although significantly greater in the EG (p < 0.05).	Q1	France
Zhu et al., 2021^31^	To examine the effects of casebased learning with STEM education concept on the clinical thinking of undergraduate nursing students.	Experimental study/ randomized experimental design with non-equivalent group pretest-posttest Total: 87 nursing study EG: 42 CG: 45	Problem-based learning program. EG: Massive Online Open Courses (MOOCs) CG: Conventional teaching	During the entire study, compared with baseline, critical thinking (275.18 and SD = 21.68), self-directed learning (215.30 and SD = 23.49), and self-efficacy (2.65 and SD = 0.45) significantly improved after implementing the intervention.	Q1	China
Nisar et al., 2022^32^	To evaluate the effectiveness of the e-training compared to conventional face-to-face training in nursing students.	Experimental study/ Single-blind, randomized controlled trial Total: 96 nursing students EG:49 CG: 47	Psychosocial management of perinatal depression training program for nursing students. EG: electronic training or e-training CG: Conventional training with specialist trainers	There was no difference in competence measured by ENACT scores between the two training methods at three months after training [M = 42.16, SD 4.85 vs. M = 42.65, SD 4.65; MD = −0.481, 95% CI; (−2.35, 1.39), p = 0.61]. No significant differences were observed between EG and CG in attitudes and beliefs regarding perinatal depression EG (p=0.22) and CG (p=0.36), self-efficacy EG (p=0.06) and CG (p=0.39), and satisfaction with training.	Q2	China
Chang et al., 2021^33^	To determine if nursing students using a mobile learning app would have significantly higher levels of knowledge about medication administration and nasotracheal suctioning, better development of skill performances on medication administration and nasotracheal suctioning, higher satisfaction, and lower cognitive load.	Experimental study/ Prospective, randomized, doubleblind, control study Total: 100 nursing students EG: 55 CG:55	Nursing activities and skills training program. EG: Virtual simulation-based mobile learning app CG: Traditional education with printed materials	After the intervention, the mean level of knowledge in the EG was higher than in the CG (t = 3.46, p < .001) with a medium effect size (d = 0.69) and good power (1−β > 0.929). Both the intrinsic cognitive load (t = −5.29, p <.001) and extraneous cognitive load (t = −6.55, p <.001) were rated significantly lower by participants in the EG than those in the CG. The mean scores of medication administration (t = 4.43, p < .001) were significantly higher in the EG than in the CG with large effect sizes (d = 0.89) and excellent power (1−β > 0.992). The mean scores of nasotracheal suctioning in the EG were significantly higher than those of the CG (t = 3.75, p <.001) with medium effect sizes (d = 0.75) and excellent power (1−β > 0.960). The EG had significantly higher satisfaction than the CG (t = 3.91, p <.001).	Q1	Taiwan
Yılmaz et al., 2022^34^	To examine the effect of the teaching method using infrared technology on PIVC success, duration, and the level of psychomotor skills and knowledge in the acquisition of PIVC skills in nursing students.	Experimental study/ group randomized controlled study 224 nursing students EG: 115 CG: 109	Peripheral intravenous catheter placement program. EG: Infrared technology CG: Traditional placement of peripheral intravenous catheters	The level of knowledge of the groups increased similarly. The EG's means scores were 60.73 ± 20.88 on the pre-test and 75.52 ± 13.21 on the post-test, while the CG's average was 60.59 ± 19.29 on the pre-test and 76.69 ± 11.12 on the post-test. The total mean PIVC skill score was significantly higher in the EG than in CG (34.13 vs. 31.88).	Q1	Turkey
Jang & Suh, 2022^35^	To develop a mobilebased multimedia Nursing Competency Evaluation (NCE) system based on the Attention, Relevance, Confidence, Satisfaction model and verify its effectiveness.	Mixed-method study/Randomized controlled study Total: 60 nursing students EG: 30 CG: 30	Nursing competency evaluation system. EG: Mobile devicebased multimedia system CG: Conventional text-based evaluation	The EG (4.6 ± 0.4) showed an average total score for effectiveness that was significantly higher than that of the CG (4.2 ± 0.5; t = −3.295, p = .002). There was no statistically significant difference in the scores for the mobile-based test between the two groups (28.6 ± 3.3 and 27.7 ± 3.7, respectively; t = −0.996, p = .324). Learning satisfaction was significantly higher in the EG (4.3 ± 0.5) than in the CG (3.8 ± 0.6; t = −3.282, p = .002).	Q1	South Korea
Rueda et al., 2022^36^	To analyze the effectiveness and perceived satisfaction in a cohort of health sciences students of non-face-to-face teaching with passive training versus face-to-face teaching with active training in the proper donning and doffing of personal protective equipment (PPE) in a clinical simulation scenario.	Experimental study/Randomized controlled trial Total: 142 students (46 nursing students and 96 physiotherapy students) EG: 71 CG: 71	PEE training program. EG: non-face-toface PEE teaching with passive training and multimedia system CG: face-to-face PEE teaching with active training in simulation scenarios.	The level of satisfaction was significantly higher in the CG (9.46 (0.78) versus EG 8.81 (1.66); p = 0.004). Conventional teaching and the use of simulation were more effective for task and procedure compliance than the EG (2.23 (1.99) vs. 1.53 (1.78); p = 0.029).	Q2	Spain
Chang et al., 2022^37^	To explore the application mode of Chatbot technologies and their effectiveness in nursing education.	Quasi-experimental study Total: 32 nursing students EG: 16 CG: 16	Educational program that aims to guide students to practice anatomy knowledge during teaching activities. EG: Knowledge-based Chatbot system CG: Conventional teaching using images and videos	The EG (Mean = 87.90; SD =11.33) had a better academic performance than the CG (Mean = 62.32; SD = 14.95). The EG (Mean = 4.07; SD = 0.65) had better critical thinking than the CG (Mean = 2.83; SD = 0.68). The EG (Mean = 4.19; SD = 0.72) had better learning satisfaction when compared with the CG (Mean = 2.83; SD = 0.68).	Q1	Taiwan
Jiménez et al., 2021^38^	To evaluate the effects of virtual simulation-based training on developing and cultivating humanization competencies in undergraduate nursing students.	Quasi-experimental study with one-group design 60 undergraduate nursing students	Basic healthcare at patients’ homes. Virtual simulation	Statistically significant differences were obtained in emotional understanding and selfefficacy dimensions, as well as in total score for the humanization scale applied, obtaining large effect sizes in all of them (rB = 0.505, rB = 0.713, and rB = 0.508, respectively). The dimensions of optimism, sociability, and affection showed slight improvements but no significant changes.	Q2	Not reported
Hwang et al., 2022^39^	To investigate the effectiveness of a virtual patient-based social learning approach to nursing education.	Quasi-experimental study with pretest-posttest design Total: 40 nursing students EG: 20 CG: 20	Physical assessment course. EG: Virtual simulation CG: Conventional teaching	In terms of learning achievement, students in the EG had a mean score of 83 (SD = 10.20), while students in the CG had a mean score of 64 (SD = 15.76). Regarding self-efficacy, EG (mean =4.54; SD =0.51) scored higher than CG (mean =3.42; SD =0.48). The EG had significantly higher post-test scores than the control group with t = 3.16 (p < .05; d = 1.01).	Q1	Taiwan
Chen et al., 2020^40^	Test the effectiveness of a program in meeting its learning outcomes among first-year nursing students.	Experimental study/ Randomized controlled trial Total: 209 nursing students EG: 99 CG: 110	Comprehensive health assessment program. EG: Simulation and educational videos. CG: Conventional teaching	In comparison with CG, EG students had significantly higher scores on knowledge [F (2,1) = 4.21, p = 0.016, η2 = 0.04], confidence [F (2, 1) = 3.57, p = 0.03, η2 = 0.03] and health assessment skills [F (2, 1) = 4.61, p = 0.004, η2 = 0.06]. Nevertheless, there were no significant differences in intention to learn and empathy between the two groups.	Q1	Singapore
Rodríguez et al., 2022^41^	To test the effectiveness of an AR-based methodology for teaching-learning aspects of the nursing curriculum (leg ulcer care), as well as to describe how AR influences different learning determinants of nursing students.	Quasi-experimental non-randomized study (TREND) Total: 137 nursing students EG: 72 CG: 65	Course on leg ulcer care. EG: Augmented reality CG: Conventional teaching	The EG participants obtained better scores in the knowledge and skills test (M = 6.08; SD = 2.26) than the CG (M = 5.23; SD = 2.38). In EG, the learning experience was rated highly: “Attention and motivation” dimension (M = 3.27; SD = 0.41), “Autonomous work” dimension (M = 3.12; SD = 0.62), and “Comprehension” dimension (M = 3.00; SD = 0.54).	Q1	Spain
Lo et al., 2022^42^	To explore the effectiveness of immersive virtual reality in improving nursing students' learning outcomes compared to the conventional learning model.	Experimental study/ prospective randomized controlled trial Total: 107 nursing students EG: 54 CG: 53	Training in nasogastric tube feeding. EG: Immersive virtual reality CG: Conventional teaching with conventional 2D video.	The paired t-test results revealed that after the intervention, the knowledge scores of both groups increased significantly: the EG from 7.75 to 8.85 (t = −6.48, p < 0.001) and the CG from 7.35 to 8.72 (t = −5.45, p < 0.001), but the between-group difference did not reach statistical significance (t = −0.54, p > 0.05). The cognitive load and satisfaction were both rated significantly higher in the EG than in the CG (t = 2.335 and t = 2.297, respectively, both p < 0.05), with medium effect sizes (d = 0.456 and 0.458, respectively). Motivation was significantly higher in EG than in the CG (t = 2.298, p < 0.05), with a medium effect size (d = 0.453).	Q1	Taiwan
Grech & Grech, 2021^43^	To compare undergraduate nursing students’ evaluations of a gamified educational webinar to a non-gamified version.	Quasi-experimental study Total: 49 nursing students EG:24 CG: 25	Webinar on determinants of health. EG: Gamified webinar CG: Non-gamified webinar	Educational quality was perceived as “good” to “very good”, in both groups. Most participants in the gamified webinar group remarked that gamification helped to increase their engagement and interaction.	Q3	Malta
Elzeky et al., 2022^44^	To assess the effects of using gamified flipped classrooms on the Fundamentals of Nursing students’ skills competency and learning motivation.	Experimental study/ Randomized controlled design with a pre-test and post-test Total:128 nursing students EG:64 CG: 64	Fundamentals of Nursing Course. EG: Gamified flipped classroom CG: Conventional flipped classrooms	A significant difference in the students’ selfconfidence (p = 0.021), skills knowledge (p < 0.001), intensity of preparation (p < 0.001), and motivation (p < 0.001) was observed between the two groups; however, no difference in the students’ skills performance (p = 0.163) was observed between the two groups after using gamified flipped classrooms.	Q1	Egypt

*EG: experimental group CG: control group*

### Cognitive and psychomotor competencies

In studying the impact of technological environments on the development of cognitive and psychomotor competencies in nursing, it is evident that several studies^19^^, ^^23^ have evaluated the impact of simulation on the acquisition of nursing competencies, showing its effectiveness in evaluation processes, information generation, decision making, knowledge and problem solving. Other applications included CPR training using intelligent technology with immediate feedback. It has also been used to manage chemotherapy extravasation and transfer of psychomotor skills to patient care^24^. However, some studies found no significant differences in knowledge levels when using simulation to recreate standardized patients and when using drug simulation^22^^, ^^23^.

The use of virtual reality (VR) and augmented reality (AR) to evaluate the effect of educational materials on knowledge and skill levels in injection practices has also been reported^25^. Yildiz & Demiray^26^ studied the effect of using VR for intravenous catheterization and fluid administration. In the first study, scores for persistence in learned knowledge and skills were higher in the EG, while in the second study, results showed higher performance of the EG's catheterization and fluid administration skills. The usefulness of VR in nursing education is highlighted as it provides tools to experience situations in a safe and realistic manner^25^^, ^^26^.

On the other hand, gamification has gained relevance in online training, demonstrating its positive impact on educational aspects. For example, Kahoot usage has been shown to improve knowledge and assessment of IM injection skills^27^. Similarly, improved learning achievement has been reported with the integration of online games for sputum suction skill training^28^. Avşar et al^.(^^29^ found significant differences between pre-and post reinforcement achievement scores using Gimkit in the EG. However, the study by Blanie et al^.(^^30^ concludes that there was no significant difference between the two groups in clinical reasoning self-assessment scores.

According to the findings of Zhu et al^.,(^^31^ the use of MOOCs improves learning and critical thinking. In contrast, e-training does not show significant differences in improving knowledge and skills compared to conventional training^32^. At the same time, Chang et al^.(^^33^ indicate that using a learning app improves learning and cognitive load. In addition, Yilmaz et al^.(^^34^ reported that using infrared technology for teaching PIVC significantly improved practical skills, while knowledge levels increased similarly in the groups. Jang and Suh^35^ also highlight the greater effectiveness of using a mobile device-based multimedia system to explore how technology can enhance clinical competency evaluation. Finally, Rueda et al^.(^^36^ evaluated the effects of non-face-to-face teaching using a multimedia system and face-to- face teaching using simulation to follow up PPE protocols and reported that conventional teaching using simulation was more effective for task completion. On the other hand, Chang et al^.(^^37^ studied the use of Chatbot technologies in anatomy teaching and observed improvements in academic performance and critical thinking.

### Affective competencies

Exploring the impact of various educational technologies on the development of affective competencies in nursing reveals a number of promising results and challenges for practice. For example, simulation has been shown to improve satisfaction, confidence, and stress reduction^19^^, ^^22^^, ^^23^ while promoting the development of humanization, emotional understanding, and self-efficacy^38^. Simulation was also used to evaluate a virtual patient-based approach to improve performance, self-efficacy, and communication^39^. However, the study by Chen et al^.(^^40^ reported that the use of simulations and videos in a program focused on health and empathy showed no significant differences between groups.

In addition, incorporating AR into the teaching of leg ulcer care has positively impacted autonomous learning, attention, and motivation^41^. Kurt and Öztürk^25^ also point out that using AR in training injection practices increases motivation to learn and self-confidence. Another study examined the impact of VR on NG tube feeding skills and found increased extrinsic motivation and satisfaction^42^.

Gamification has been observed to have a positive impact on self-efficacy, motivation, learning engagement, and satisfaction^28^^, ^^30^; it also increases engagement and interaction and promotes self confidence and motivation development^44^. In addition, the use of MOOCs improves self-efficacy^31^, while the use of mobile learning, multimedia systems, and chatbots improves student satisfaction^33^^, ^^35^^, ^^37^. However, it is evident that e-training does not show significant differences in improving self-efficacy, attitudes, beliefs, and satisfaction with training compared to conventional training^32^.

## Discussion

Research highlights the importance of educational technology in developing cognitive and psychomotor competencies in nursing education. The results support the effectiveness of simulation in a safe and controlled environment^45^ for these competencies development^19^^, ^^24^. However, while it may improve students' knowledge and practical skills, some studies report that transferring these skills to the actual clinical setting may be limited^24^. On the other hand, VR and AR are promising, significantly improving knowledge and skill levels^25^^, ^^26^, and are innovative and effective in creating immersive, realistic, and effective learning environments^46^.

Gamification is emerging as an effective pedagogical strategy in nursing education^47^. Its implementation has demonstrated significant improvements in knowledge and practical skills^27^^, ^^29^. However, while it seems to have a positive impact on certain areas of learning, no significant differences were observed on tests of clinical reasoning skills^30^.

It also shows that the use of MOOCs^31^, mobile simulation applications^33^, infrared technology^34^, multimedia systems^35^^, ^^36^, and chatbots^37^ significantly improves knowledge, practical skills, and critical thinking, supporting the idea that technology plays a key role in transforming nursing education by providing new opportunities for autonomous learning, guided practice, and personalized feedback.

The impact of simulation on affective competencies is highlighted, supporting its effectiveness in various dimensions of learning^19^^, ^^22^^, ^^23^^, ^^38^^, ^^40^ that reaffirm the humanization of care and respect for the human being^48^. Although they found no significant differences in the achievement of empathy^40^, the results suggest that the simulation increases satisfaction, confidence, emotional understanding, self-efficacy, and communication, and reduces stress. On the other hand, VR and AR impact the development of emotional skills^49^ and are effective in reducing fear^25^ and developing nursing skills^41^^, ^^42^. The benefits observed in terms of cognitive load, satisfaction, and motivation support the feasibility of its integration into nursing education programs to improve the quality of learning and prepare students for the challenges of practice. However, more research is needed to understand the underlying mechanisms better and to optimize the design and implementation of these technologies.

Regarding gamification, it not only improves academic performance, but also strengthens engagement, motivation, and learning satisfaction. These environments promote a more interactive and relevant learning experience that encourages emotional and affective development. Although it was observed that there were no significant differences in self-efficacy and satisfaction with the use of e-training^32^, other approaches such as MOOCs^31^, mobile learning applications^33^, multimedia assessment systems^35^^, ^^36^ and the use of artificial intelligence^37^ have been shown to promote a more interactive and relevant learning experience that contributes to the emotional and affective development of students. It is important to consider that although student satisfaction is crucial, effectiveness in applying practical skills in clinical settings is also critical to developing affective competencies such as self-confidence and self-efficacy in professional nursing practice.

The results obtained provide a diverse view of the impact of different technologies on developing nursing competencies. The relevance of educational technology in this field is highlighted, allowing its contribution to be recognized beyond the instrumental level, seeking to improve the quality of human life, enhancing unique skills, and fostering the essential abilities to become sensitive to the problems of others^50^. Although strengths that enrich learning and professional practice are highlighted, limitations and areas for further research are also identified. The capacity of these technologies to provide immersive and realistic learning environments that can enhance the acquisition of knowledge and skills is remarkable. However, there is a need to deepen the competencies that promote the humanization of care.

It is crucial to consider the diversity of contexts in which these technologies are used. It is essential to understand how technological tools can be adapted and effectively applied in Latin American contexts, where cultural and socio-economic factors can significantly influence their implementation and effectiveness. Despite the evidence for the use of technology in nursing education, there is a need to address its limitations and conduct additional research to better understand its impact and maximize its benefits, especially at the affective level.

## Conclusion

Research on the use of technology in nursing education highlights the positive impact on cognitive and psychomotor competencies. However, essential aspects of humane care, such as empathy, creativity, and understanding of the human being, are lacking. It is essential that nursing education focuses not only on technical and cognitive competencies but also on the development of affective competencies that enable compassionate, empathetic, and respectful communication with others to ensure comprehensive, person-centered care.
